# Alcohol-related liver disease: also a question of what you drink?

**DOI:** 10.37349/edd.2023.00022

**Published:** 2023-06-30

**Authors:** Finn Jung, Victor Sánchez, Annette Brandt, Ina Bergheim

**Affiliations:** Department of Nutritional Sciences, Molecular Nutritional Science, University of Vienna, A-1090 Vienna, Austria

**Keywords:** Beer, wine, spirits, ethanol, resveratrol, hop

## Abstract

Excessive alcohol intake is still among the leading causes of chronic liver diseases. Epidemiological studies suggest that per capita consumption of alcohol from various alcohol beverages e.g., beer, wine, or spirits, differs markedly between different areas of the world. Studies further suggest that different alcoholic beverages may impact the development of alcohol-related liver diseases (ALD) differentially. Specifically, results of several more recent epidemiological studies suggest that consumption of wine and herein especially of red wine may be less harmful in relation to the development of liver diseases than the intake of hard spirits. Results of studies evaluating the effects of beer on the development of ALD in humans are rather contradictory. Here, results of studies assessing the impact of wine, beer, and spirits on the development of ALD as well as possible underlying mechanisms are summarized and discussed.

## Introduction

Alcohol intake is still among the leading causes of death and health impairments in the world. Indeed, in 2020, 1.03 billion males (35.1% of the male global population aged ≥ 15 years) and 312 million females (10.5% of female global population aged ≥ 15 years) consumed alcohol in amounts exceeding the non-drinker equivalent (NDE) [1], the latter being defined as the level of alcohol consumption at which the risk of health loss for a drinker is equivalent to that of a non-drinker. Epidemiological data further suggest that in 2020, ~1.78 million [95% uncertainty interval (UI) 1.39–2.27] of global deaths among males aged 15–49 years were related to alcohol intake [1]. Furthermore, ~5.1% of the global burden of disease and injury is attributable to alcohol consumption [1–3]. The liver is among the organs thought to be most vulnerable with respect to heavy alcohol intake and resulting damage [4]. Herein heavy alcohol use is mostly defined as 3 and more standard drinks per day for women (~40 g of ethanol per day for women) and 4 and more standard drinks per day for men (~50–60 g of ethanol per day for men) [5]. Indeed, it has been shown that ~27.3% of the deaths attributed to cirrhosis in men and 20.6% of deaths attributed to cirrhosis in women are related to alcohol consumption [6, 7] and that a daily intake of 31–60 g of ethanol may already increase the odds by 10.9% to develop liver diseases [8–10]. However, it has also been reported that not only the amounts of ethanol consumed may impact the development of alcohol-related liver diseases (ALD) but that also the type of alcoholic beverage consumed may impact the development of liver damage. Starting from this background, the aim of the present review is to summarize findings regarding the role of different alcoholic drinks with a focus on non-distilled drinks on the development of ALD and to highlight some of the underlying molecular mechanisms.

### Alcohol intake in various world regions

**Figure 1:**
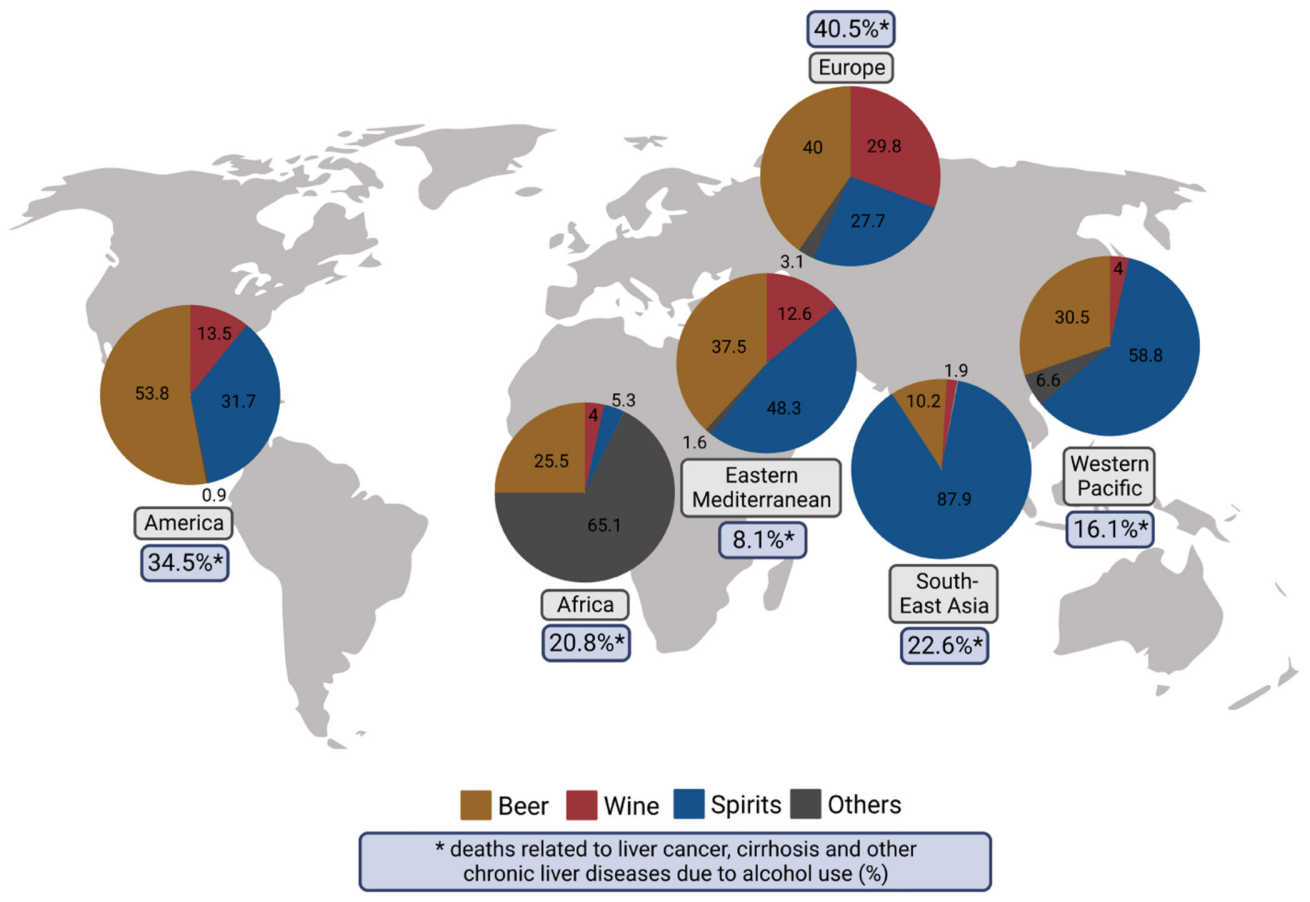
Recorded alcohol per capita consumption (%) of different alcoholic beverages and deaths related to liver cancer, cirrhosis, and other chronic liver diseases related to alcohol use (%) separated by World Health Organization (WHO) region. Calculation of deaths related to liver cancer, cirrhosis, and other chronic liver diseases due to alcohol use (%) by dividing the number of deaths related to liver cancer, cirrhosis, and other chronic liver diseases due to alcohol use with the number of overall deaths related to liver cancer, cirrhosis, and other chronic liver diseases obtained from the Global Burden of Disease study results tool [74, 75] which is maintained by the Institute for Health Metrics and Evaluation. Data based on [2] and [74] and figure created with Biorender.com

Studies suggest that the amounts of ethanol consumed vary considerably between different geographical regions [1, 2]. For instance, in North America, but also many European and Asian countries as well as Russia, intake of ethanol ranges from 10–13 L per person per year, whereas in the Eastern Mediterranean Region and across North Africa, alcohol consumption is reported to be markedly lower with being even close to zero in some countries [2]. Similar to the findings regarding total amounts of ethanol consumed, intake of different alcoholic beverages also varies considerably among different regions and even more so countries [2]. Indeed, when regarded globally, hard spirits are the type of alcoholic drink consumed the most (~44.8%), while wine accounts for ~11.7% of the ingested alcohol and beer for ~34.3% [2]. However, when being stratified by regions, in the South East Asian Region, hard spirits account for ~87.9% of the consumed alcohol-containing beverages, while beer and wine only contribute 10.2% and 1.9%, respectively, to the overall alcohol intake [2]. In contrast, in the European Region, hard spirits only make up for 27.2% of the consumed ethanol while wine and beer contribute to 29.8% and 40.0%, respectively, of the consumed ethanol [2] (see also Figure 1). There are also considerable differences found in the consumption pattern within the different regions. For instance, while in France and Italy, ~60% of the consumed alcoholic drinks are consumed as wine and herein especially red wine, in Germany, Austria, and the Czech Republic, wine only accounts for ~30% of the ingested alcoholic drinks. In these countries, epidemiological data suggests that beer intake contributes to ~54% of the ingested alcohol [2].

### Stages of ALD and ethanol metabolism

**Figure 2:**
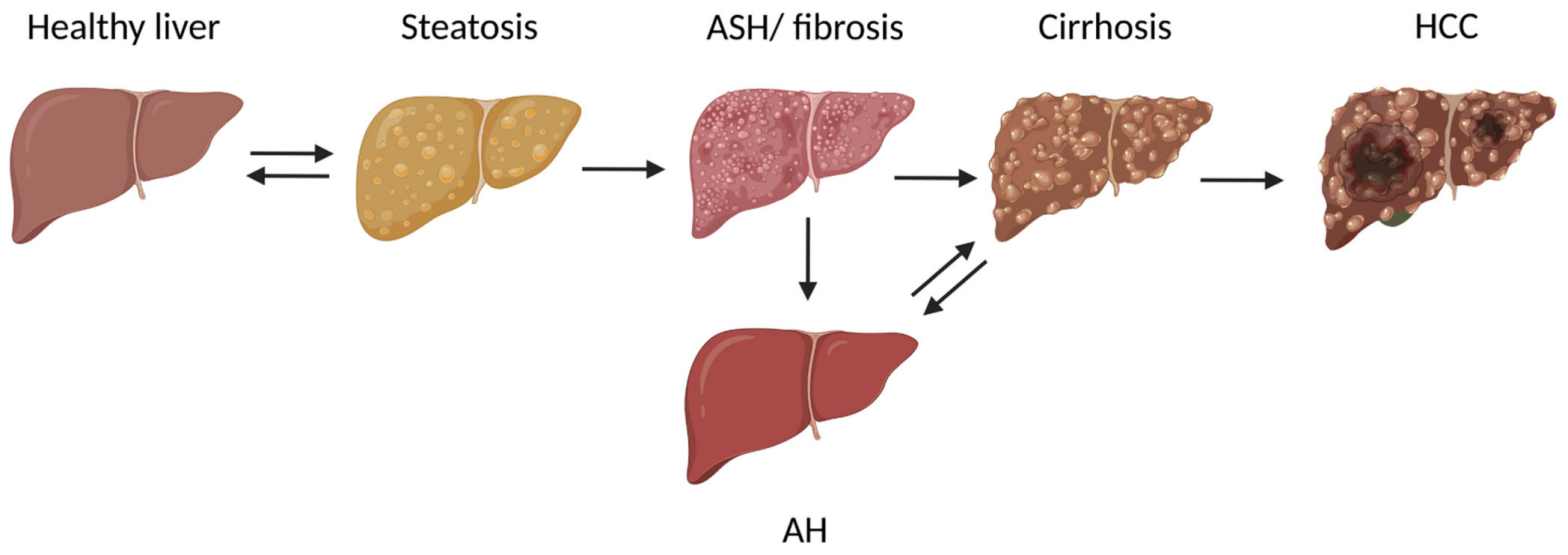
Progression of ALD. ALD comprise a wide spectrum of diseases ranging from simple steatosis (fatty liver) to AH, ASH, or fibrosis, and cirrhosis as well as in some cases, even HCC. Figure was created based on [76] with Biorender.com

ALD comprise a wide spectrum of diseases ranging from simple steatosis (fatty liver) to alcoholic hepatitis (AH), alcoholic liver fibrosis (ALF), and cirrhosis as well as in some cases, even hepatocellular carcinoma (HCC, for overview see [6] and see Figure 2). However, results of epidemiological studies suggest that only about 25–35% of chronic heavy drinkers develop severe forms such as AH or cirrhosis [11]. Thus, ALD can be classified as asymptomatic or early ALD [fatty liver and alcoholic steatohepatitis (ASH)] or advanced ALD (defined as AH, cirrhosis, and its complications such as ascites, portal hypertension-related bleeding, hepatic encephalopathy, sepsis, multi-organ dysfunction, and HCC) [5, 12].

### Ethanol uptake and metabolism

Due to its physio-chemical properties, ethanol is rapidly taken up by passive diffusion in the small intestine and reaches the liver from there through the portal vein. Ethanol metabolism in the gastrointestinal tract is low; still, ethanol metabolizing mucosal enzymes [isoforms of the alcohol dehydrogenase (ADH) see below] in the stomach contributes to the so-called first-pass effect [13]. Studies have shown that gastric ADH activity is lower in women than in age-matched men. This, at least in part, may explain the gender differences in regard to tolerance and damage related to alcohol intake found when comparing men and women consuming similar doses of ethanol [13]. Furthermore, studies revealed that not only gender but also ethnicity influences alcohol tolerance and subsequently drinking pattern (for overview see [14]). Genetic association studies showed that mutations in alcohol metabolizing enzymes [ADH and aldehyde dehydrogenase (ALDH)] are highly prevalent in Asia while being almost absent in people of European or African origin [15]. For instance, it is estimated that ~45% of East Asians (Japanese, Chinese, Koreans) carry a mutation in the ALDH2*2 allele (*Glu504Lys, rs671*) encoding for an inactive ALDH2 [16]. These genetic variants influence alcohol oxidation rate resulting in the so-called ‘flushing syndrome’ and have therefore been suggested to be associated with a lower risk to develop alcohol dependence [17]. Furthermore, the alcohol elimination rate varies considerably between species. For example in rodents, being broadly used in *in-vivo* settings investigating the effects of acute and chronic ethanol exposure, alcohol metabolism is up to 5 times faster compared to humans [18]. Therefore, while leading to severe drunkenness and maybe even death in humans, alcohol concentrations up to 6 g/kg bodyweight are well tolerated by mice and rats and are therefore commonly used in experimental settings employing these rodents [19, 20].

**Figure 3:**
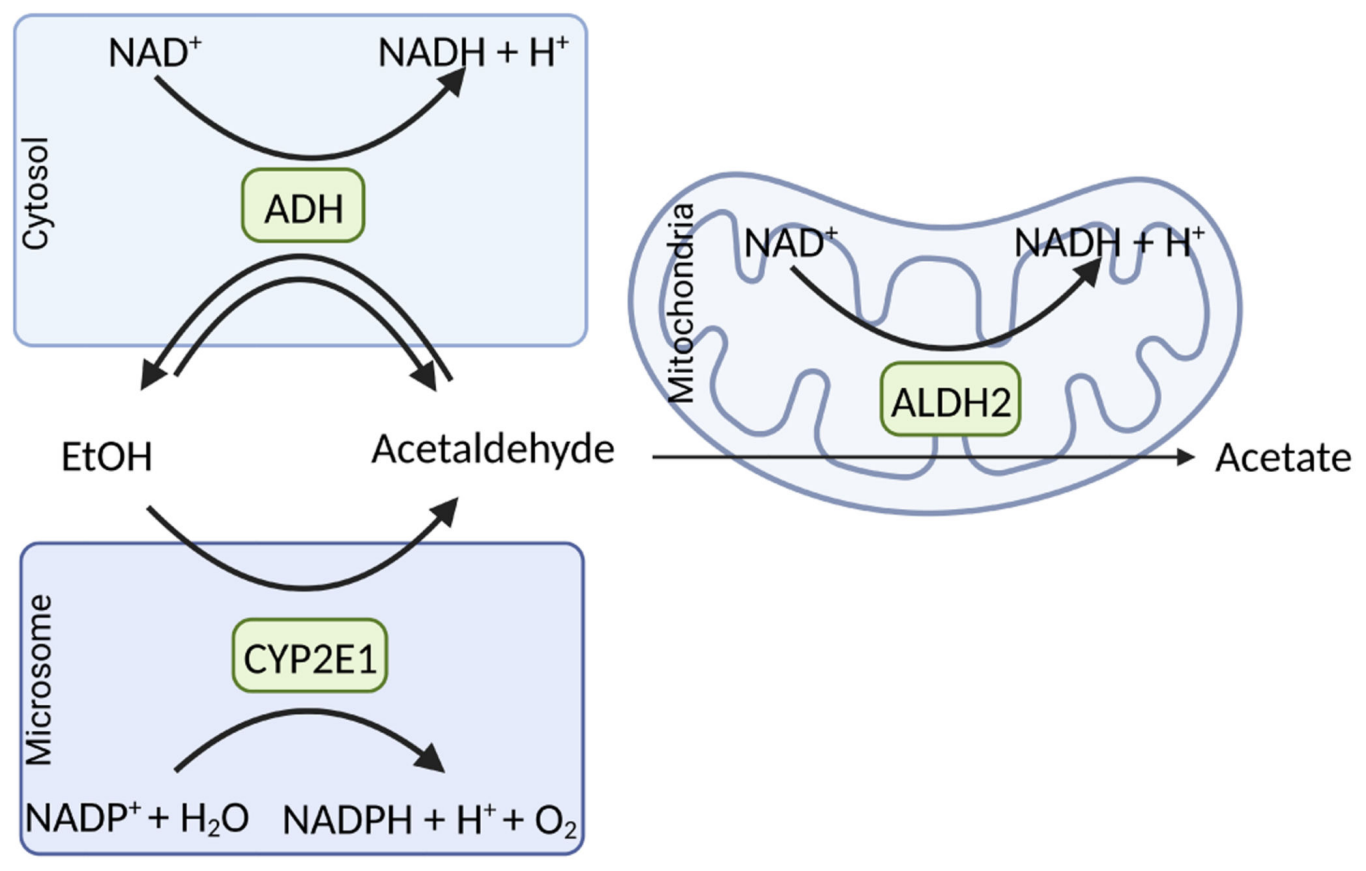
Major oxidative pathways of alcohol metabolism. In the cytoplasmic compartment of the cell, the oxidation of ethanol to acetaldehyde is catalyzed by the ADH being associated with the reduction of nicotinamide adenine dinucleotide (NAD^+^) to NADH + H^+^. CYP2E1, an enzyme with a high abundance in microsomes, is involved in alcohol elimination to acetaldehyde when alcohol concentrations are high. In this reaction, nicotinamide adenine dinucleotide phosphate (NADP^+^) and H_2_O are needed to form NADPH and H^+^ + O_2_. The formed acetaldehyde is oxidized mainly by ALDH2 allocated in the cell’s mitochondria, which results in the formation of acetate and NADH + H^+^. Figure was created based on [77] with Biorender.com. EtOH: ethanol

Over 80% of the imbibed ethanol is metabolized by hepatocytes in the liver [21]. There are several enzymes that can metabolize ethanol to acetaldehyde: 1) the cytosolic ADH, 2) cytochrome P450 enzymes and herein predominantly cytochrome P450 2E1 (CYP2E1) but also other cytochromes like CYP1A2 and CYP3A4 allocated in the microsomes [22], as well as 3) catalase (CAT), an enzyme found in peroxisomes (for overview see [23] and see Figure 3). The latter enzyme system is of minor importance for ethanol metabolism in the liver, whereas studies suggest that it may contribute to a larger extent to ethanol elimination in the brain [24].

### ADH

**Figure 4:**
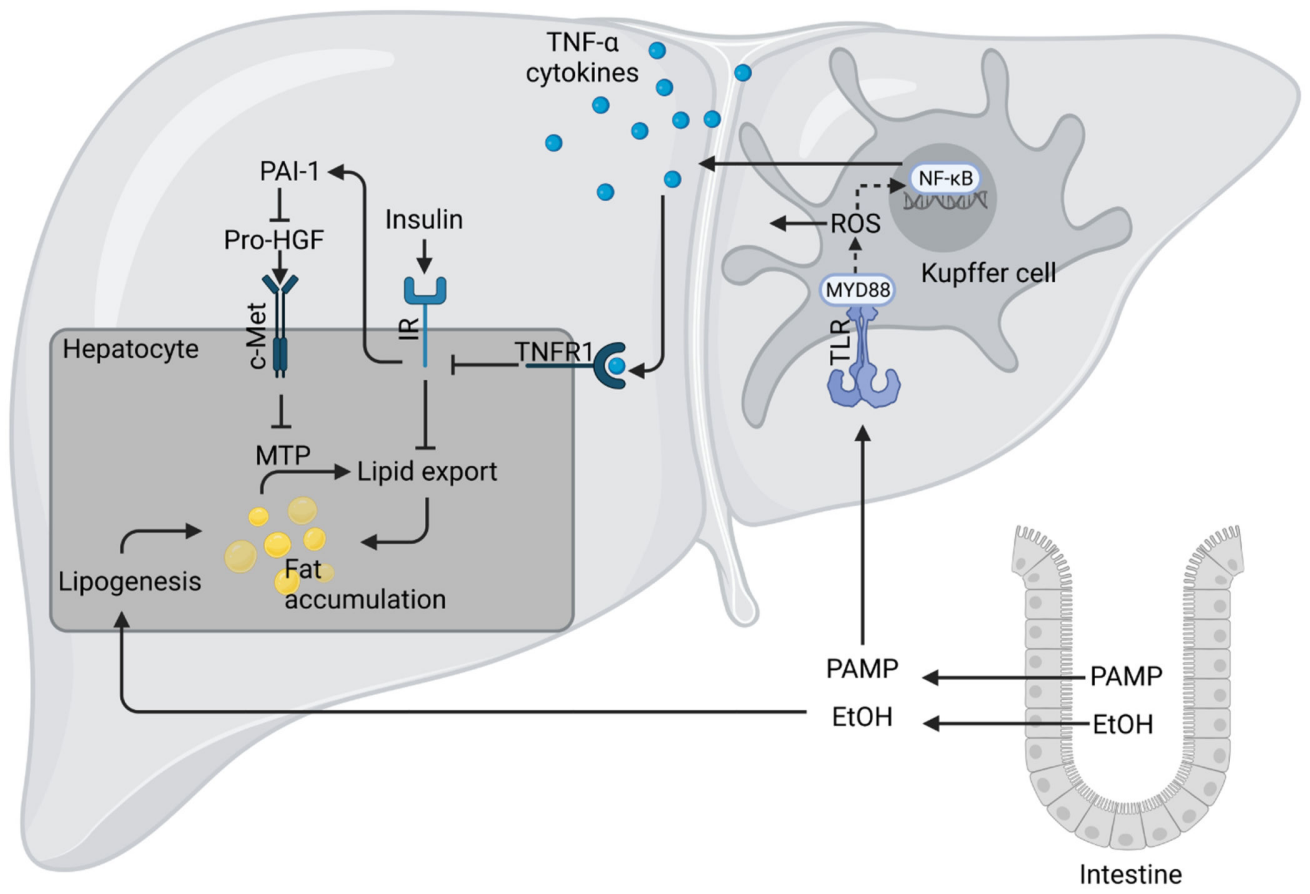
Mechanism of ALD. High and/or chronic alcohol intake (EtOH) can cause intestinal dysbiosis and an increased translocation of PAMPs, and herein especially lipopolysaccharide (LPS). Upon reaching the liver through portal circulation, LPS or other PAMPs are recognized by the TLR4 or other TLRs mostly found on Kupffer cells and initiates an inflammatory response through myeloid differentiation primary response 88 (MYD88) and inducible nitric oxide synthase (iNOS)-dependent signaling cascades. This results in the activation of nuclear factor-κB (NF-κB), release of reactive oxygen species (ROS) and pro-inflammatory cytokines like tumor necrosis factor (TNF)-α. TNF-α has been shown to impair insulin signaling and through plasminogen activator inhibitor (PAI)-1-dependent signaling cascades to alter lipid export through hepatic growth factor (HGF)/mesenchymal-epithelial transition factor (c-Met) and microsomal triglyceride transfer protein (MTP)-dependent pathways. In addition, through its ADH-dependent metabolism, ethanol can also add to an enhanced lipogenesis. Figure was created based on [78] with Biorender.com. IR: insulin receptor

The oxidation of ethanol to acetaldehyde through ADH and subsequently to acetate by ALDH requires NAD^+^ as a cofactor, which during chronic, heavy drinking results in a reduction of the ratio of NAD^+^ to NADH + H^+^. The damaging effect of ethanol on the liver has been attributed to this shift in redox equivalents including the metabolic shift toward fatty acid synthesis and increased production of lactate [21, 25]; however, as briefly discussed below, and elsewhere described in detail, other mechanisms like a change in intestinal microbiota composition and increased translocation of pathogen-associated molecular patterns (PAMPs, e.g., bacterial endotoxin) and subsequently an activation of toll-like receptor (TLR)-dependent signaling pathways may also be critical herein (for overview see [26] and Figure 4).

### CYP2E1

CYP2E1 along with some other CYPs has been shown to contribute to ethanol metabolism [27]. CYP2E1 oxidizes ethanol in the presence of molecular oxygen (O_2_) to acetaldehyde along with the formation of NADPH + H^+^ and H_2_O [28]. The substrate specificity of CYP2E1 for ethanol is higher when compared to ADH while its catalytic efficiency is lower. Still, as this enzyme is inducible and its half-life has been shown to be extendable, CYP2E1 contributes markedly to ethanol elimination in settings of chronic high ethanol intake [22, 29] (see Figure 3). However, CYP2E1 can also produce ROS, which have been linked to the development of ALD (for overview also see [30]).

### CAT

CAT, an enzyme located in the peroxisomes detoxifying peroxide (H_2_O_2_) has also been shown to oxidize ethanol to acetaldehyde under the use of peroxide forming H_2_O as a side product. As already mentioned above and reviewed by others in more depth [22], in the liver, this enzyme is of minor importance for the elimination of ethanol.

## Alcoholic beverages and the liver: the impact of spirits, wine, and beer intake on the liver

The alcohol-attributable burden of disease is usually assessed under the assumption that the type of alcoholic drink consumed does not matter. Risk relations so far are mostly based on doses of raw ethanol consumed and patterns of use rather than in terms of the type of alcoholic drink ingested (for overview also see [31, 32]). And while many epidemiological studies suggest that alcohol intake affects disease development in a dose-response manner following a J-shaped curve [33, 34], results of some epidemiological studies assessing the effects of alcohol on the development of ALD also suggest that herein the type of alcoholic beverage consumed e.g., whether a person is a wine, beer, or spirits drinker or maybe a “mixed consumer” might be important, too. For instance, in the study of Mitchell et al. [35], enrolling subjects with a mean daily alcohol intake of 20 g over the last 18 years, it was reported that exclusive wine drinkers especially when consuming wine in a non-binge pattern, had lower mean fibrosis stages and lower odds of advanced fibrosis [odds ratio (OR) 0.20, 95% confidence interval (CI) 0.06–0.60, *P* = 0.01] when compared to lifetime abstainers. Interestingly, in this study, similar effects were not found for beer drinkers, further suggesting that not only the absolute amount of alcohol ingested but also the type of beverage consumed (e.g., wine, beer, or spirits) may impact the effect of alcohol consumption on the liver. However, when interpreting the data of the study of Mitchell et al. [35], one has to consider, that alcohol doses consumed were rather moderate than high. Still, somewhat in line with these findings, a study conducted on 219,279 Norwegian men also reported that wine consumption was associated with a reduction of the hazard ratio (HR; HR 0.68, 95% CI 0.46–1.00) for liver-related mortality [36]. Similar effects were not reported for the consumption of hard spirits and beer, respectively; however, again average alcohol intake was rather moderate than high. Indeed, wine drinkers consumed 1.07 units/day while spirit and beer drinkers consumed 1.25 units/day and 1.17 units/day, respectively, and intake differed between wine, beer, and spirit drinkers (here: 1 unit = ~17 g ethanol). Results of the Danish Cancer, Diet, and Health study (1993–2011) also suggest that men whose alcohol consumption mainly consisted of wine had a lower HR than men who drank no or little alcohol as wine [37]. Indeed, in this study, it was shown that in men who drank 14–28 drinks/week, the HR compared to those drinking little, some, and mostly wine, respectively, was 7.47 (95% CI: 1.68; 33.12), 3.12 (95% CI: 1.53; 6.39), and 1.69 (95% CI: 0.79; 3.65) [37]. Others, studying 43,242 Swedish men, also reported a trend toward a reduced risk for severe liver disease in men consuming > 50% of alcohol as wine (HR 0.79, 95% CI 0.48–0.102, *P* = 0.06); however, the trend was no longer found when a multivariable model was applied [38]. In the study of Stokkeland et al. [39], it was reported that women with alcoholic liver cirrhosis have consumed 14,010 drinks during their lifetime compared to 27,440 drinks in women with alcohol dependence without liver cirrhosis. Interestingly, in the same study, women with alcohol dependence without alcohol cirrhosis were reported to have consumed 11,350 glasses of beer while those with alcohol cirrhosis had only drunk 1,611 glasses of beer. Similar differences in drinking patterns were not found in men or in wine and spirits [39]. Ye et al. [40] in 2011 also reported differences regarding beverage-specific effects on cirrhosis mortality rates in the US. In earlier studies of Kerr et al. [41], they performed a pooled cross-sectional time series analysis of per capita total alcohol, beer, wine, and spirits consumption and all-cause cirrhosis mortality with data from Australia, Canada, New Zealand, the United Kingdom, and the United States, and found an association of cirrhosis mortality with spirit consumption rather than with beer or wine intake. Indeed, in this study, it was reported that a 1 L increase in spirits consumption is associated with a 35.2% increase in cirrhosis mortality rate. Results of a recent prospective cohort study of UK Biobank participants excluding abstainers and individuals with previous cancers, liver cirrhosis, and stroke as well as myocardial infarcts suggested that spirit drinking as well as beer and cider drinkers were at higher risk of liver cirrhosis (spirits: HR 1.48; 95% CI 1.08–2.03; beer/cider: 1.36; 95% CI 1.06–1.74) compared to red wine drinkers after adjusting for the average weekly alcohol consumption [42]. Interestingly, while lowering the relative risk (RR) of all-cause mortality, consuming alcohol with food, it did not have an association with the risk of liver cirrhosis. In this study, it was also shown, that daily alcohol intake was associated with a higher liver cirrhosis risk with no further distinguishment regarding type of alcoholic drink [42]. Results of a recently published analysis focusing on the Mediterranean diet and fatty liver risk in overweight older Italians revealed that individuals with a fatty liver index > 60 had a higher intake of wine [43] and consumed alcohol at a higher percentage over an extended period of time. These data further suggest that wine consumption and herein especially at higher levels may also confer adverse effects on the liver. In the prospective UK Million Women Study, no differences between the type of alcoholic beverage consumed e.g., wine *vs*. all other alcoholic beverages with respect to the incidence of liver cirrhosis was found. However, the number of women who only consumed beer without spirits intake was low and the intake of the latter was often afflicted with confounders like smoking or living in a deprived area [44]. Interestingly, in this study, women who usually consumed their alcoholic drinks with a meal had a lower RR (RR 0.69, 95% CI 0.62–0.77; *P* < 0.0001) for cirrhosis than those who consumed alcohol without a meal despite comparable total alcohol intake between both strata [44]. Differences between the latter study and the others discussed above might have resulted from gender-specific differences. Indeed, in most other studies, no or only a limited number of women were enrolled often prohibiting a valid assessment of the impact of different alcoholic beverages on the development of ALD in women. Taken together, further studies are needed to settle the question if consumption of non-distilled alcoholic drinks like beer and wine and distilled alcoholic beverages like vodka, whiskey, and schnapps impacts the liver differently. For overview of average alcohol consumption in studies discussed in this chapter see Table 1. Also, it has to be kept in mind that regardless of the outcome of these studies, the main ingredient in the alcohol-attributable burden is ethanol. Indeed, while some compounds found in non-distilled alcoholic beverages may even bear some beneficial effects on the liver (e.g., xanthohumol and iso-α-acids derived from hops and found in beer, or resveratrol found in red wine, see below), other organs may not benefit from these compounds.

## Possible mechanisms underlying the less harmful effects of (red) wine and beer in regard to the development of ALD

**Figure 5:**
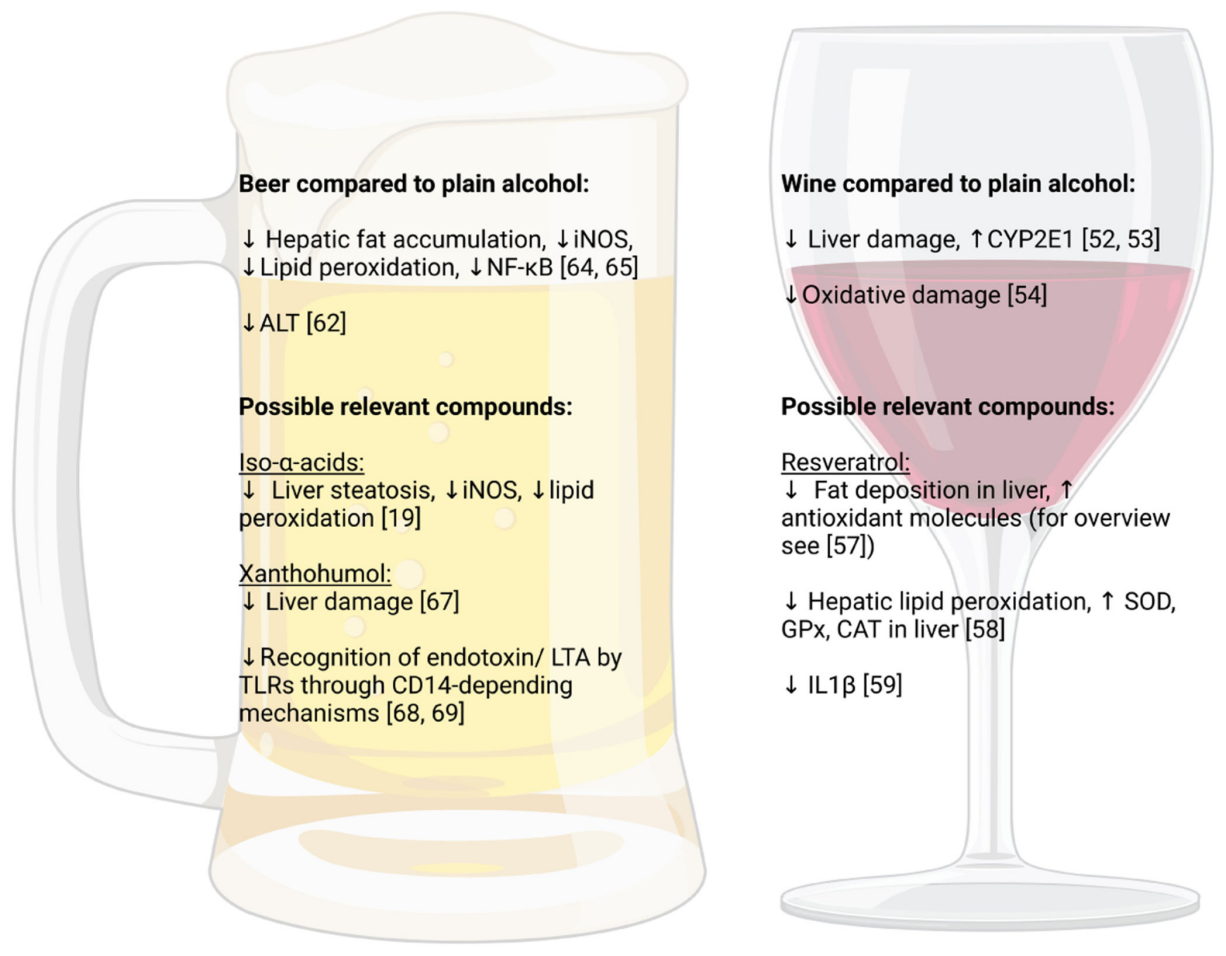
Possible molecular mechanisms associated with potential beneficial effects of wine and beer over other types of alcohol. Figure created with Biorender.com. ALT: alanine aminotransferase; CD14: cluster of differentiation 14; GPx: glutathione peroxidase; IL: interleukin; LTA: lipoteichoic acid; SOD: superoxide dismutase. ↓: reduction; ↑: increase

As summarized above, even when consumed chronically at elevated amounts, non-distilled alcoholic beverages, and herein especially red wine, seem to bear less harm to the liver than the consumption of spirits. Next to other well-studied factors involved in the development and progression of ALD e.g., changes in lipid metabolism and the production of ROS resulting from the metabolization of ethanol as well as a hepatic overload of iron (for overview see [45]), several possible mechanisms are discussed that may add to this ‘less’ harmful effect of non-distilled alcoholic beverages on the liver. For instance, it has been shown that moderate wine drinkers appear to be at lower risk to become heavy drinkers [46, 47], which may add to the explanation of some of the reported beverage-specific morbidity and mortality findings. Furthermore, both wine and beer are not only composed of ethanol but due to their production process contain also marked amounts of secondary plant compounds like polyphenols and side products produced during fermentation (for overview see Tables 2 and 3, and [48–51]). However, it needs to be kept in mind when interpreting data from epidemiological and experimental studies that not only ripeness and quality as well as the growing region of barely and hop but also grapes used for beer and wine production, respectively, may influence considerably the contents of secondary plant compounds in the end product but also that the production process may vary considerable between breweries and wineries in different regions of the world. In the following, results of some experimental studies focusing on effects of wine and beer in the context of the development of ALD are summarized. Also, some possible molecules and molecular mechanisms underlying the less damaging effects of these non-distilled alcoholic beverages are highlighted (findings also summarized in Figure 5).

### Wine

Supporting the above-summarized results of epidemiological studies, results of animal studies also suggest that wine intake and herein especially red wine consumption at moderate to high doses (e.g.,7 mL/kg body weight of a 12.5 vol% wine for 28 days) may be associated with a diminished development of liver damage and increased CYP450 enzymes activity (especially CYP2E1) than the intake of plain ethanol [52, 53]. These effects have been reported to lead to lesser oxidative damage compared to plain ethanol [54].

In wine and especially in red wine, many phenolic compounds are found, and several have been proposed to be involved in the health effect of (red) wine (for overview also see [55]). Indeed, studies enriching red wine with Vineatrol^®^, a vine-shoot phenolic extract being particularly rich in resveratrol and its oligomers, reported that the vine-shoot in combination with red wine diminished a high-fat diet plus plain ethanol-induced liver inflammation in hamsters [56]. However, despite these data suggesting that polyphenols found in (red) wine may lessen the harmful effects of plain ethanol on the liver, specific compound(s) have not yet been identified. In the following some findings related to resveratrol being thought to be one of the secondary plant compounds in red wine involved in the less harmful effects of red wine on the liver are summarized.

### Resveratrol

Results of numerous *in vitro* and *in vivo* studies now suggest that resveratrol found in red grapes but also other plants may be involved in the less damaging effects of red wine on the liver (for overview also see [57]). Indeed, results of several studies suggest that at moderate to high doses of resveratrol (10–500 mg/kg), often exceeding what is ingested even when drinking 1 L of red wine (average content 2–15 mg/L resveratrol), may reduce fat deposition in the liver and enhance expression of antioxidant molecules (for overview also see [57]). However, most studies assessing the effects of resveratrol focused on (liver) diseases unrelated to alcohol intake. For instance, it was shown that at doses of 5 g/kg diet resveratrol dampened hepatic lipid peroxidation and ameliorated the ethanol-related reduction of SOD, GPx, and CAT activity in liver [58]. In another study employing 10 mg resveratrol/mL drinking water, the polyphenol reduced alcohol-induced IL1β levels in liver as well as liver damage along with lowering mortality rates in mice [59]. Furthermore, in studies mimicking weekend drinking in combination with a high-sucrose diet, rats treated concomitantly with resveratrol (6 mg/L) were markedly protected from liver damage being again associated with a protection against the pro-oxidative state associated with the feeding regime [60]. Contrasting these findings, others reported that the addition of resveratrol in plain ethanol (150 mg/kg body weight) may even worsen the development of alcohol-related liver damage and enhanced mortality in mice which seemed at least in part be related to a greater induction of CYP2E1 [61]. And while the concentrations of ethanol solution were rather high and there were no isocaloric controls, these data suggest that 1) resveratrol may not be the exclusive compound found in red wine involved in its less damaging effect on the liver and 2) further studies are needed to determine the effects of resveratrol in setting of alcohol-related liver damage. Taken together, further studies are warranted to determine which compound(s) in (red) wine are involved in the less damaging effects on the liver compared to an iso-alcoholic consumption of similar amounts of plain ethanol.

### Beer

**Figure 6:**
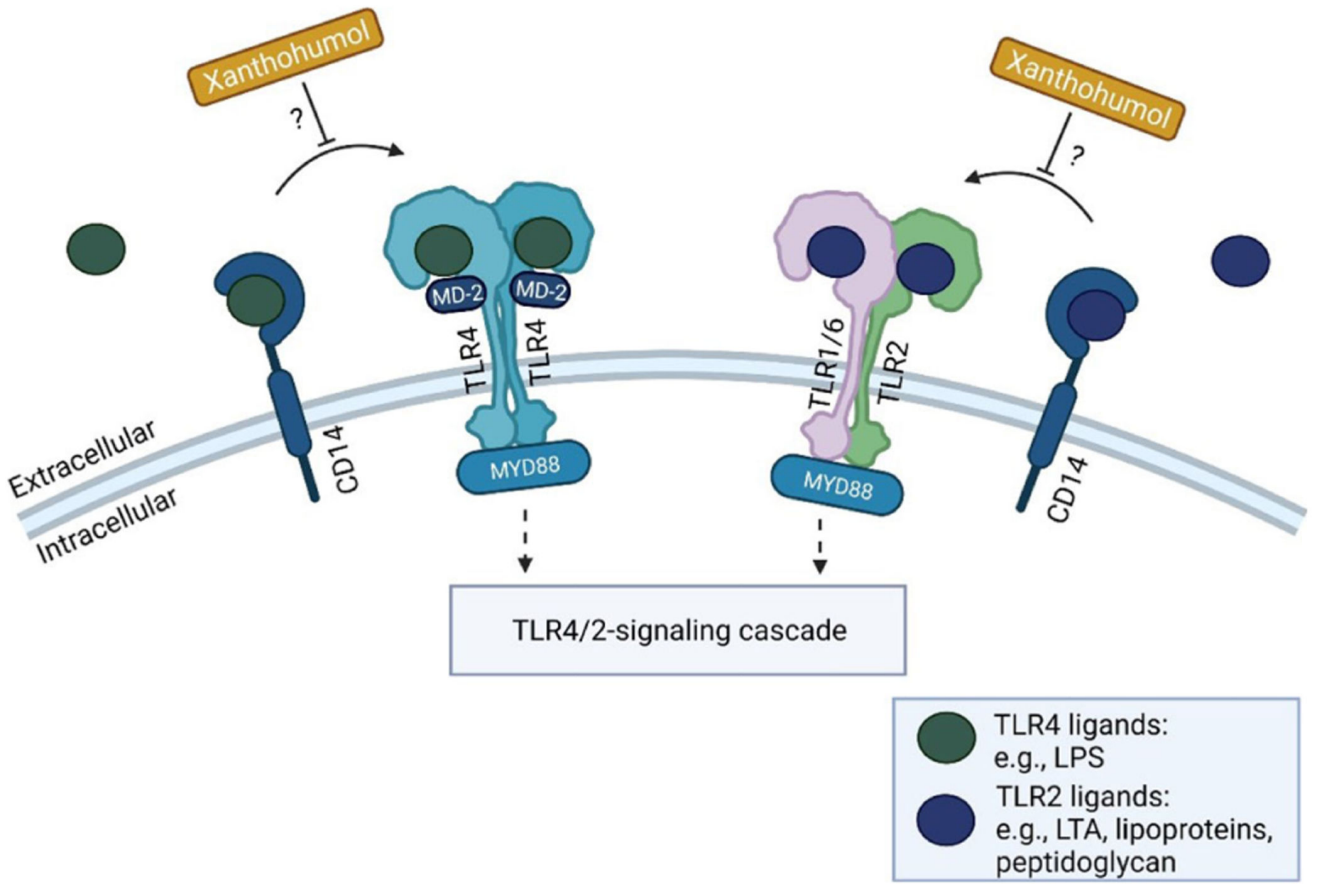
Possible inhibitory effects of xanthohumol on TLR signaling cascade. Xanthohumol may inhibit the recognition of ligands for TLR2 (e.g., LTA, lipoproteins, or peptidoglycan), and TLR4 (e.g., LPS) through CD14-depending mechanisms further affecting related signaling cascades. Figure was created based on [81] with Biorender.com. MD-2: myeloid differentiation factor-2. ?: possible mechanism

In a study in rats comparing the effects of beer, tap water enriched with a 10% alcoholic solution prepared with red wine and plain ethanol in an *ad libitum* feeding model for 6 weeks, it was shown that the chronic intake of beer was associated with no increases in transaminase activities in serum, whereas both plain ethanol and red wine resulted in a similar increase [62]; however, animals fed red wine had the highest alcohol intake and the highest total antioxidant status [63]. Results of our own group suggest that in both male and female mice, the acute intake of iso-alcoholic and -caloric amounts of beer compared to plain ethanol resulted in lower fat accumulation in the liver along with lower iNOS expression, lower markers of lipid peroxidation and less induction of NF-κB [64, 65]. Also, the intake of moderate amounts of beer (2.5 g ethanol/kg body weight) diminished the development of a high fat, high fructose, and high cholesterol diet-induced non-alcoholic fatty liver disease (NAFLD) in mice; an effect not found in mice receiving iso-alcoholic and caloric amounts of plain ethanol [66]. It was further shown in mice that effects of beer on the liver may differ between different types of beer [64]. Indeed, we showed that the ingestion of Pilsner beer being richer in secondary plant compounds like iso-α-acids and xanthohumol was afflicted with markedly fewer damaging effects on the liver than stout in mice [64]. Supporting the hypothesis that secondary plant compounds found in hop may be involved in the less damaging effect of beer on the liver compared to the intake of plain alcohol, it was further shown by us that acute consumption of beer brewed without hops was similarly harmful to the liver of mice as the intake of comparable amounts of plain ethanol [20]. Results of studies with rodents further suggest that pre-treatment of animals with iso-α-acids at concentrations similar to those taken up when consuming ~2.5 L of beer attenuated the development of acute alcohol-induced liver steatosis being associated with a lower induction of iNOS and a lessening of markers of lipid peroxidation in liver [19]. The pre-treatment of rats with xanthohumol before ingesting acutely high amounts of ethanol (4 g ethanol/kg body weight) has also been shown to be associated with markedly dampened liver damage [67]. Recently, it has been shown that xanthohumol even at concentration as low as those found when consuming 0.25–0.5 L beer, can interfere with the TLR-dependent recognition of PAMPs through interfering with CD14 [68, 69]. Specifically, it was shown that xanthohumol may through CD14-depending mechanisms inhibit the recognition of endotoxin and LTA, respectively, by TLR4 and TLR2 receptors (see Figure 6). Increases in both TLR2 and TLR4 ligands have been reported to be associated with the development of ALD in humans [70].

Taken together, results of human and animal studies suggest that hops derived compounds like iso-α-acids and xanthohumol can pass the intestinal barrier [71–73] and may in rodents and humans, even at low doses achievable with the intake of 0.25–2.5 L of beer exert some beneficial health effects including the dampening of the development of liver disease of various etiologies.

## Conclusions

While the study of the effect of alcohol intake on the development of liver diseases and associated molecular mechanisms has been the focus of many experimental, clinical, and epidemiological studies, reliable and unbiased data assessing the effect of different alcoholic drinks e.g., non-distilled *vs*. distilled alcoholic beverages are still limited. Indeed, some older and some more recent studies suggest that the intake of non-distilled alcoholic beverages and herein especially the intake of red wine may affect the liver differently than the consumption of hard spirits. However, studies also suggest that drinking pattern, gender, and whether or not the alcoholic drink is consumed with or without a meal may affect the effects of the alcohol drink of the liver. The impact of the different factors but also the compounds involved in the different effects of non-distilled alcoholic beverages *vs*. distilled alcoholic drinks on the liver have not yet been clarified. Accordingly, there is a need for studies with large and clinically well-characterized patients as well as validated assessment tools of alcohol intake to address the above-mentioned research questions. Along with these human studies, further experimental studies using doses of compounds found in alcoholic beverages achievable with a “normal” alcohol intake in humans are warranted to determine molecular mechanism underlying the different effects of alcoholic beverages on the liver but maybe also other organs. Still, even when results of cell cultures and rodents studies as well as some epidemiological studies suggest that effects of non-distilled and distilled alcoholic beverages may markedly differ with respect to liver health, it needs to be kept in mind that in other organs and the central nervous system these differences may not be prevalent, and that effects of ethanol regarding dependence and mental alteration may not be affected by the compounds found to be beneficial for the liver.

## Abbreviations

ADH: alcohol dehydrogenase
AH: alcoholic hepatitis
ALD: alcohol-related liver diseases
ALDH: aldehyde dehydrogenase
ASH: alcoholic steatohepatitis
CAT: catalase
CD14: cluster of differentiation 14
CI: confidence interval
CYP2E1: cytochrome P450 2E1
HCC: hepatocellular carcinoma
HR: hazard ratio
iNOS: inducible nitric oxide synthase
LTA: lipoteichoic acid
NAD^+^: nicotinamide adenine dinucleotide
NF-κB: nuclear factor-κB
PAMPs: pathogen-associated molecular patterns
ROS: reactive oxygen species
RR: relative risk
TLR: toll-like receptor
TNF: tumor necrosis factor

## Figures and Tables

**Table 1:** Average alcohol consumption in studies investigating the impact of spirits, wine, and beer intake on the liver

Study	Alcohol consumption
Mitchell et al. [35]	20 g (2.3–60 g) alcohol/week
Tverdal et al. [36]	1.07–1.25 drinks/day (15.6–21.0 g alcohol)
Askgaard et al. [37]	14–28 drinks/week (168–336 g alcohol)
Hagström et al. [38]	5.1–24.6 g alcohol/day
Stokkeland et al. [39]	Alcoholic cirrhosis: 4.4 drinks/day (52.8 g alcohol/day)
	Alcohol dependence: 5.8 drinks/day (69.6 g alcohol/day)
Ye et al. [40]	7.95–10.94 L alcohol/capita per year
Jani et al. [42]	8–400 g alcohol/week

**Table 2:** Concentrations of the most abundant polyphenols in beer (based on [79])

Group	Concentration
Phenolic alcohols	3–40 mg/L
Phenolic acids	10–30 mg/L
Phenolic amines and amino acids	10–20 mg/L
Flavonoids catechins	0.5–13 mg/L
Anthocyanidins	4–80 mg/L
Flavonols	< 10 mg/L

**Table 3:** Concentrations of the most abundant polyphenols in wine (based on [80])

Group	Concentration
Anthocyanidins	90–400 mg/L
Flavonols	up to 60 mg/L
Condensed tannins	1.2–3.3 g/L
Flavanones	up to 25 mg/L
Flavones	0.2–1 mg/L
Non-flavonoids	60–566 mg/L

## Data Availability

Not applicable.
